# Expiratory Muscle Strength Training in COPD Dysphagia Management: A Survey of Speech-Language Pathologists

**DOI:** 10.3390/jcm15020733

**Published:** 2026-01-16

**Authors:** Sandra Brandon, Stanislava Antonijevic, Ruth Mc Menamin

**Affiliations:** Department of Speech and Language Therapy, School of Health Sciences, University of Galway, H91 TK33 Galway, Ireland

**Keywords:** chronic obstructive pulmonary disease (COPD), dysphagia, expiratory muscle strength training (EMST), speech-language pathology, clinical practice, respiratory rehabilitation, Ireland

## Abstract

**Background:** Chronic Obstructive Pulmonary Disease (COPD) affects over 400 million people worldwide. Ireland reports the highest COPD-related mortality and hospitalizations in Europe. Dysphagia impacts approximately 50% of people with COPD (PwCOPD) and contributes to COPD exacerbations, hospitalizations, and mortality. Expiratory Muscle Strength Training (EMST) improves respiration and swallowing for PwCOPD; however, little is known about its clinical use by Speech-Language Pathologists (SLPs). **Methods:** A cross-sectional online survey, developed in accordance with CHERRIES and CROSS guidelines, aimed to explore SLPs awareness, assessment approaches, treatment protocols, training, and confidence in EMST delivery. It was distributed to SLPs working with adults with dysphagia in Ireland. Purposive and snowball sampling were used, with a target sample size of *n* = 258. **Results:** The response rate was 36% (*n* = 92). Awareness of EMST was high (99%, *n* = 91). 53% (*n* = 49) reported using EMST. Among EMST users, 20% employed objective assessments of maximal expiratory pressure (MEP), while most calibrated devices to 75% of MEP and followed the “rule of fives” treatment protocol. 29% had formal training in EMST. SLPs with ≤10 years’ clinical experience and those working in acute hospitals used EMST most often. Confidence was influenced by training, experience, access to specialized respiratory equipment, and interdisciplinary team members. **Conclusions:** SLPs EMST awareness is high, but implementation practices remains variable, with low uptake of formal training and limited use of objective MEP assessment. Findings highlight the need for structured training and population-specific protocols to support consistent and confident EMST delivery for PwCOPD.

## 1. Introduction

Chronic Obstructive Pulmonary Disease (COPD) is a progressive inflammatory lung disorder characterized by persistent airflow limitation and frequent exacerbations [1]. Internationally, COPD affects over 400 million people, with prevalence projected to reach 600 million by 2050 [2]. COPD is the third leading cause of death worldwide [3]. Ireland reports the highest rate of respiratory-related death and hospitalization for COPD exacerbations in the European Union [4,5]. Exacerbations, characterized by an acute worsening of dyspnea, cough, or sputum production, are strongly associated with reduced survival [6]. Preventing and effectively managing exacerbations is therefore a key priority within both Irish and international healthcare systems [7,8].

Dysphagia (eating, drinking, and swallowing impairment) is increasingly recognized as a significant comorbidity in COPD, affecting up to 50% of people living with the disease (PwCOPD) [9]. Dysphagia is associated with increased risk of aspiration pneumonia, recurrent exacerbations, hospitalization, and increased mortality [10,11]. PwCOPD with aspiration-related pneumonia experience poorer outcomes than those with community-acquired pneumonia [12]. Timely identification and management of dysphagia is therefore critical in reducing adverse respiratory events. Dysphagia in PwCOPD is multifactorial, involving impairments in swallow safety, efficiency, and sensory function. Altered respiratory–swallow coordination, characterized by inspiration immediately before or after swallowing, rather than the typical exhale–swallow–exhale pattern, significantly increases aspiration risk [13]. Oral and pharyngeal muscle weakness contributes to prolonged bolus transit and pharyngeal residue [11,14], while diminished laryngopharyngeal sensitivity predisposes PwCOPD to silent aspiration [15], which occurs in up to one-third of this population [16,17]. Speech-Language Pathologists (SLPs) play a central role in the management of dysphagia [18]. In PwCOPD, however, Speech-Language Pathology (SLP) interventions are often compensatory (e.g., diet modification, altered feeding strategies, and fatigue management) rather than rehabilitative [19,20]. Rehabilitative interventions such as Expiratory Muscle Strength Training (EMST), which target the physiological mechanisms underlying dysphagia, are increasingly recommended for PwCOPD [19,21,22]. Improving airway protection is one important goal of dysphagia therapy to reduce aspiration of food and liquid into the lungs [18]. Evidence is growing in support of EMST to improve airway protection, cough and swallow function in neurological dysphagia [23,24], the elderly population [25], and in other conditions that cause impaired respiratory function, such as Parkinson’s disease [26,27].

EMST is a non-invasive intervention utilizing a handheld, pressure-threshold device with an adjustable one-way valve that provides graded resistance during exhalation, thereby strengthening expiratory and submental muscles [28,29]. Training protocols typically begin with assessment of maximal expiratory pressure (MEP). MEP is the highest pressure that can be developed during a forceful expiratory effort against an occluded airway [30]. It can be tested using the EMST device or by objective measures using a handheld digital manometer (e.g., MP01 Micro Respiratory Pressure Meter; Micro Direct, Inc., Lewiston, ME, USA) or standardized testing by a respiratory physiologist [31]. The device’s resistance setting is then calibrated to a percentage of the individual’s MEP, which serves as the therapeutic target. Progression involves incremental increases in resistance to induce muscle overload of the expiratory and submental muscles [28].

EMST improves respiratory muscle strength, cough effectiveness, and airway protection in PwCOPD [22,32,33,34,35,36], and its inclusion in pulmonary rehabilitation programs has been recommended [20,37]. A meta-analysis indicated that PwCOPD who underwent EMST achieved significantly greater improvements in swallowing outcomes than controls (effect size = 1.39) [22]. Additionally, a randomized, double-blind clinical trial involving individuals with Parkinson’s disease reported that a four-week EMST program significantly reduced pharyngeal residue, thereby enhancing swallowing efficiency [26]. Collectively, these findings underscore EMST as a feasible and effective dysphagia intervention for PwCOPD.

Despite this growing evidence base, little is known about the extent to which SLPs use EMST in clinical practice with PwCOPD or the factors influencing its implementation. As part of understanding current practice, it is important to consider the educational and professional context in which SLPs are introduced to EMST. In Ireland, neither the SLP professional body, the Irish Association of Speech and Language Therapists (IASLT) nor the statutory regulatory body, CORU, specifies EMST as a required clinical competency for qualification or registration. CORU’s Standards of Proficiency [38] and IASLT standards for dysphagia management [39] outline broad requirements for dysphagia assessment, e.g., oral-motor assessment and management, e.g., postural maneuvers during swallowing; however, EMST is not explicitly included within these standards. In Ireland, SLPs determine individually whether to pursue formal or self-directed EMST as part of their continuing professional development (CPD). Translating research-based interventions into clinical settings is known to be variable; even well-supported evidence-based treatments are often inconsistently or only partially integrated into practice [40]. To date, only one study, conducted in North America, has explored SLPs’ (*n* = 48) use of EMST for PwCOPD and dysphagia [21]. In that study, 71% of respondents (*n* = 34) agreed that PwCOPD benefit from EMST, and it was ranked as the fifth most frequently used dysphagia treatment with this population. However, beyond North America, the extent to which EMST is adopted by SLPs as a dysphagia intervention for PwCOPD has not been explored.

A further consideration in EMST implementation is treatment protocol selection. Studies exploring the impact of EMST on swallow function in Parkinson’s disease report calibrating the EMST device to 75% of MEP, and completing five sets of five exhalations daily for five days per week for five weeks—the “rule of fives” protocol [27,28,41]. Modifications to this protocol have been carried out with people with amyotrophic lateral sclerosis [42]. A randomized sham-controlled trial, conducted by Plowman and colleagues [42], found that reducing the pressure-threshold/resistance of the device to 50% of MEP and extending treatment duration to eight weeks demonstrated improvements in respiratory and swallow function in amyotrophic lateral sclerosis populations [42]. To date, no consensus exists on the optimal EMST protocol for PwCOPD. In addition, there is a lack of evidence to guide SLPs on how MEP should be measured (via the EMST device versus objective measures) with this population.

Given Ireland’s high burden of COPD-related morbidity and mortality, this study investigated the clinical practices of SLPs working in Ireland with PwCOPD and dysphagia, with a specific focus on EMST implementation as a dysphagia intervention. Data collection occurred during the COVID-19 pandemic, when EMST was classified as an aerosol-generating procedure [43], and PwCOPD were considered at high risk of severe infection [44]. A secondary aim was therefore to examine whether pandemic-related restrictions affected EMST delivery in clinical settings. These findings are important to inform preparedness for this vulnerable population in the event of future global viral respiratory pandemics.

## 2. Materials and Methods

### 2.1. Study Design

A cross-sectional survey design was employed to investigate SLPs’ awareness, implementation practices, self-reported confidence ratings, and training when using Expiratory Muscle Strength Training (EMST) for dysphagia management with PwCOPD in Ireland. The study was conducted and reported in accordance with the Checklist for Reporting Results of Internet E-Surveys (CHERRIES) [45] and Consensus-Based Checklist for Reporting of Survey Studies (CROSS) [46] guidelines for survey research.

### 2.2. Participant Recruitment

Qualified SLPs registered with CORU (Ireland’s national health and social care professionals’ regulator) and currently working with adults with diagnoses of COPD and dysphagia across acute, outpatient, community, and rehabilitation settings were eligible to participate. SLPs working exclusively with pediatric populations were excluded.

Non-probability purposive sampling [47] was used to target SLPs who met the inclusion criteria, with additional recruitment through snowball sampling [48] to increase participation. The estimated target population was based on CORU’s register of 1964 SLPs, of whom approximately 40% (*n* = 785) were estimated to work with adults. A sample size of 258 was calculated using the Qualtrics survey sample size calculator software (Qualtrics XM Platform, Copyright © Qualtrics, Provo, UT, USA. https://www.qualtrics.com/articles/strategy-research/calculating-sample-size, version accessed on 8 August 2021), with a population size *n* = 785, margin of error = +/− 5%, confidence level = 95%, and standard deviation estimate SD = 0.5. These figures were based on information available at the time of the study from the statutory body (CORU), where registration is mandatory for SLPs who wish to practice in Ireland. Membership of professional bodies is elective, not mandatory in Ireland, so CORUs’ registration list was the most representative source available.

The open, online survey was hosted on Microsoft Forms and disseminated via professional networks, including the Irish Association of Speech and Language Therapists (IASLT), IASLT Research Special Interest Group (SIG), Adult Dysphagia SIG, the Irish Speech and Language Therapy Managers Group, and the Independent Speech and Language Therapists of Ireland (ISTI). In the absence of a national register detailing the demographics of the target population, it was not possible to identify a single gatekeeper for participant recruitment. Instead, the aforementioned professional networks were selected as the most likely to reach SLPs working with adults with dysphagia across diverse clinical contexts and geographical regions. Gatekeepers within these organizations disseminated study information, including the survey link, and circulated the questionnaire (see Supplementary S2). Recruitment was conducted over a three-month period (November 2021–January 2022) and was supplemented by advertisements on social media platforms (e.g., Facebook and X) and promotion at professional study events, such as the Dysphagia SIG Winter Study Day.

Survey completion was anonymous, on a voluntary basis, and did not include remuneration. Although IP addresses were not tracked to maintain anonymity, measures were taken to reduce duplicate responses, including primarily distributing the survey via selected gatekeepers, instructing respondents to submit only once, and reviewing completion times and response patterns for duplicates.

To maximize completeness of responses, the survey was set up to ensure that all questions were answered in each section before the respondent could proceed to the next section. To encourage participation, respondents could opt into a draw for a complimentary EMST 150 device (Aspire Products, LLC, Cape Carteret, NC, USA). Entry into this draw required submission of a contact email address. This email address was used solely for contacting the winner of the EMST 150 device and was not linked to participant responses. To further promote participation, reminder emails were issued by gatekeepers at weeks five and eight following the initial survey dissemination.

### 2.3. Questionnaire Development

Following a review of the literature, it was determined that no validated survey questionnaire existed on this topic. Based on this literature review, including a meta-analysis and systematic review [22], a multi-disciplinary expert committee designed a questionnaire in accordance with the CROSS [46] and CHERRIES [45] criteria.

A pilot study [49], was conducted with fourteen SLPs who worked across acute hospitals, outpatient, and rehabilitation settings. SLPs were recruited from the Republic of Ireland (*n* = 4) and Northern Ireland (*n* = 10). They had varying levels of clinical experience working with PwCOPD (ranging from one to twenty-five years). Inclusion criteria also required participants to have familiarity with the delivery of EMST to PwCOPD and dysphagia. Pilot study participants reviewed the questionnaire and provided feedback on the feasibility, content validity, and reliability of the tool. Revisions were made based on feedback regarding content adequacy, question clarity, and survey flow. Test–retest reliability was evaluated with two SLPs, one week apart, demonstrating high reliability (Pearson’s r = 0.85).

The final instrument, “Expiratory Muscle Strength Training in COPD dysphagia management: A survey of Speech-Language Pathologists (SLPs) in the Republic of Ireland”, (Supplementary S1), comprised 17 mandatory questions (13 closed questions and 4 open-ended questions) within three sections: (i) participant information and consent; (ii) demographic and caseload profile; and (iii) awareness, clinical practices, and confidence ratings in EMST use with PwCOPD. Closed questions included binary and multiple-choice formats, while open-ended items allowed respondents to include free text about their practices and confidence when delivering EMST to PwCOPD and dysphagia. The number of characters and words permitted for open-ended questions was not restricted. To enhance response rates and reduce burden on respondents, adaptive questioning was included in the survey design [45].

### 2.4. Ethical Considerations and Informed Consent

The study was reviewed and approved by the University of Galway, Research Ethics Committee (Approval code: 2020.08.001). Respondents provided informed consent electronically prior to survey commencement. Participation was voluntary. Respondents could withdraw at any time before survey submission. No identifying data were collected for the purposes of data analysis.

### 2.5. Data Protection and Duplicate Entry Prevention

All data were collected anonymously through Microsoft Forms and stored securely on a password-protected institutional server. Neither IP addresses nor personal identifiers were recorded. To prevent duplicate entries, responses were manually screened for similarities in timestamps, demographics, and professional profiles. Only unique, complete entries were retained for analysis.

### 2.6. Data Analysis

Quantitative data were exported to Microsoft Excel and IBM SPSS Statistics (Version 28.0, IBM Corp., Armonk, NY, USA) for analysis. Descriptive statistics (frequencies, percentages) were used to summarize categorical and ordinal variables. Chi-square tests examined associations between demographic factors (years of experience, work setting, caseload composition) and EMST practices and confidence ratings.

Responses to open-ended survey questions (#9, #11, #14, and #16) were exported into Nvivo (Lumivero, version 14) [50] for content analysis [51]. Given the limited evidence on SLPs’ use of EMST with PwCOPD in Ireland, an inductive content analysis approach was adopted [51,52,53]. This approach [51] allowed categories to emerge directly from the data while enabling quantification through frequency counts [54]. Analysis followed three phases: preparation, organizing, and reporting. In the preparation phase, responses were read repeatedly to gain familiarity, and individual sentences were identified as units of analysis. During the organizing phase, open coding was performed to label responses, which were then grouped into categories and higher-order headings through iterative comparison and reflection. Nvivo [50] generated frequency counts for each category and subcategory, and the third author independently verified the coding to achieve consensus, ensuring rigor and reliability in the analysis.

## 3. Results

The results of this study are presented across the following subsections: survey response; demographics; assessment practices using EMST; treatment protocol regimens; training undertaken by SLPs; self-reported confidence ratings; content analysis of perceived factors that may improve confidence using EMST; and the impact of COVID-19 on EMST use.

### 3.1. Survey Response

Of an estimated target sample size of 258 eligible SLPs, 92 completed the online survey, yielding a response rate of 36%. Awareness of EMST by respondents was high (99%, *n* = 91), and a lower 53% (*n* = 49) reported using EMST with PwCOPD and dysphagia. All 49 respondents who used EMST with PwCOPD completed every question with no missing data, and these responses were included for further data analysis.

### 3.2. Respondent Demographics

Demographics of respondents (*n* = 49) who reported using EMST with PwCOPD are summarized in Table 1 and Table 2.

Most respondents worked in acute hospital settings (65.3%, *n* = 32), followed by outpatient/community care (18.4%, *n* = 9) and rehabilitation services (14.3%, *n* = 7). Clinical dysphagia experience ranged from 0 to >20 years, with the largest proportion having 6–10 years of experience (28.6%, *n* = 14). PwCOPD represented between 1% and 50% of respondents’ clinical caseloads. SLPs working in acute settings (65.3%, *n* = 32) reported using EMST more frequently than those in non-acute settings (34.7%, *n* = 17), although this association was not statistically significant (χ^2^ (9) = 12.33, *p* = 0.195). The association between years of clinical experience and EMST use reached borderline significance (χ^2^ (4) = 9.71, *p* = 0.046). This pattern suggests that SLPs with ≤10 years of practice may be more inclined to implement EMST in clinical work with PwCOPD and dysphagia, but this finding should be interpreted with caution. Caseload composition was not a significant determinant of EMST use (χ^2^ (2) = 0.44, *p* = 0.802); SLPs whose clinical caseload consisted of a low percentage of PwCOPD (1–25% caseload) were as likely to use EMST as SLPs whose caseloads contained a larger percentage of PwCOPD (25–50% caseload).

### 3.3. Assessment Practices Using EMST

Most respondents (96%, *n* = 47) assessed Maximum Expiratory Pressure (MEP) using the EMST device, while 20% (*n* = 10) used objective measures. Among those using objective measures to test MEP, 8% (*n* = 4) used a hand-held digital manometer and 12% (*n* = 6) referred PwCOPD to a respiratory physiologist. SLPs who referred to a respiratory physiologist worked exclusively within acute care hospital settings. Years of experience were significantly associated with MEP assessment method (χ^2^ (32) = 47.97, *p* = 0.035), with less experienced (≤5 years’ experience) relying more on EMST devices, and those with >15 years’ experience more frequently using objective measures. Caseload composition was inversely related to assessment method (χ^2^ (16) = 29.07, *p* = 0.023); SLPs with fewer PwCOPD on their caseload were more likely to use objective measures.

### 3.4. Treatment Protocol Regimens

Most SLPs (85.7%, *n* = 42) followed the protocol based on the “rule of fives”, i.e., 5 repetitions × 5 sets per day, 5 days per week for 5 weeks at 75% of MEP. A smaller proportion (14.3%, *n* = 7) reported modifying this protocol based on patient fatigue, motivation, or clinical complexity. Protocol modifications included lowering pressure-threshold/resistance (25–50% MEP), fewer daily repetitions, or extended program duration. Choice of protocol was not significantly associated with clinical experience (χ^2^ (24) = 29.14, *p* = 0.215), work setting (χ^2^ (54) = 49.26, *p* = 0.657), or caseload profile (χ^2^ (12) = 5.42, *p* = 0.943).

### 3.5. Training Undertaken by SLPs

Formal EMST was undertaken by 29% (*n* = 14) of respondents, while 71% (*n* = 35) relied on informal learning (peer-instruction and/or self-directed). Of those formally trained, 25% (*n* = 12) attended face-to-face workshops, and 4% (*n* = 2) completed online courses. Training was not significantly associated with work setting (χ^2^ (126) = 149.02, *p* = 0.079) or clinical experience (χ^2^ (58) = 67.53, *p* = 0.139).

### 3.6. Self-Reported Confidence Ratings

Respondents rated their confidence in using EMST with PwCOPD (Table 3). Most respondents (69%, *n* = 35) reported moderate confidence (scores: 6–8/10 or 60–80% confidence rating), 27% (*n* = 13) rated low confidence (scores: ≤5/10 or ≤50% confidence rating) and 4% (*n* = 2) had high confidence (scores: ≥9/10 or 90% confidence rating). Confidence was not associated with work location (χ^2^ (45) = 28.11, *p* = 0.977), clinical experience (χ^2^ (36) = 31.35, *p* = 0.689), or caseload composition (χ^2^ (18) = 19.65, *p* = 0.353).

### 3.7. Perceived Factors Reported to Improve SLP Confidence in EMST Delivery

Respondents were asked to describe how their confidence could be improved when delivering EMST to PwCOPD. All respondents (*n* = 49) answered this survey question. Content analysis of open-ended responses revealed four key categories: Training, Knowledge, Experience/Exposure, and Resource Access (Table 4).

### 3.8. Impact of COVID-19

Twenty percent (*n* = 10) suspended EMST during the COVID-19 pandemic due to its classification as an aerosol-generating procedure. Most (80%, *n* = 39) continued EMST under infection control protocols, with 6% (*n* = 3) using telehealth exclusively and 14% (*n* = 7) adopting hybrid delivery.

## 4. Discussion

This study offers novel insights into clinical practices, confidence, and training needs of SLPs using EMST to treat dysphagia in PwCOPD. Several key factors emerged from the findings: awareness and usage patterns, assessment and treatment protocols, training and confidence, and the impact of the COVID-19 pandemic.

### 4.1. Awareness and Usage Patterns

The finding that most respondents were aware of EMST as a dysphagia treatment for PwCOPD is encouraging, as awareness is a critical precursor to clinical adoption [55]. However, awareness alone does not guarantee implementation [56]. While over half of respondents (53%) reported using EMST, this level of uptake indicates that significant gaps remain in embedding EMST as a standard intervention for PwCOPD and dysphagia. Similar patterns have been observed in other allied health contexts, where evidence-based practices fail to achieve widespread adoption without targeted implementation strategies [57].

Implementation of EMST was more common among SLPs working in acute hospitals, which may be reflective of increased interdisciplinary working in acute settings and easier access by these respondents to interdisciplinary specialists (e.g., respiratory physicians, respiratory physiologists), highlighting the importance of integrated care pathways [58]. The higher adoption of EMST in dysphagia clinical practice with PwCOPD by early-career clinicians suggests that recent graduates may act as “early adopters” of evidence-based interventions, whereas more experienced clinicians may rely on established routine and biases towards certain therapy interventions that they have found successful in the past [59], a trend consistent with Rogers’ Diffusion of Innovations theory [60]. This trend may also reflect the relatively recent emergence of EMST as a dysphagia intervention, meaning that more experienced clinicians may not have received formal education about this approach during their undergraduate professional training. This highlights the need for targeted continuing professional development (CPD) to ensure consistent uptake across all experience levels [61]. The observed association between years of clinical experience and EMST may be confounded by work setting, as a greater proportion of respondents were based in an acute hospital where access to specialized interdisciplinary teams for PwCOPD is more readily available. Notably, EMST use was not influenced by the proportion of PwCOPD on clinicians’ caseloads, suggesting that barriers are systemic rather than patient specific.

These findings have clear clinical and policy implications: professional bodies should prioritize formal training in EMST within CPD frameworks, and healthcare services should develop standardized protocols and resource allocation strategies to support implementation. Without such measures, EMST risks remaining an underutilized intervention for PwCOPD despite evidence of its efficacy [22].

### 4.2. Assessment and Treatment Protocols

Assessment of MEP was predominantly conducted using the EMST device rather than objective measures (such as a digital handheld manometer or standardized testing by a respiratory physiologist). The survey did not explore why clinicians relied on device-based estimation, and although this reliance may reflect a pragmatic response to systemic barriers faced by SLPs [62], qualitative data describing perceived factors that enhance confidence in EMST delivery (Table 4) provide some valuable context. Respondents highlighted limited access to manometry and reliance on respiratory physiologists (“Resource Access” category), as well as a need for further supervision and technical training (“Training” category). These categories align with our findings that respondents who referred to PwCOPD for a physiologist-led assessment were working in acute hospitals. These findings reflect the broader evidence that interdisciplinary access and collaboration are essential for consistent COPD care pathways [58]. Limited use of objective measures to test MEP may hinder the collection of robust pre and post-treatment data, reducing opportunities for single-case research and clinical trials, and consequently limiting publication and evidence generation. Addressing this gap is essential; future policy should prioritize resource allocation and interdisciplinary collaboration to ensure objective MEP assessment becomes routine practice. Furthermore, this area warrants systematic investigation to strengthen research capacity and promote high-quality evidence for EMST in PwCOPD.

In terms of treatment protocol, most respondents set the treatment dosage as 75% of MEP and implemented a therapy protocol using the “rule of fives” (completing five sets of five exhalations daily for five days per week for five weeks) [28]. These treatment practices are consistent with published research on EMST use with other conditions that impair respiratory function [27,28]. However, to date, there is no established specific treatment protocol for PwCOPD. Protocol modifications were made by a minority to accommodate patient-specific factors such as fatigue, which underscores the need for flexible clinical guidelines tailored to PwCOPD complexity. There was no significant association between protocol variations and clinician experience or work setting, suggesting a broad consensus on core EMST parameters but limited standardization in individualized adjustments.

These findings underscore the importance of developing standardized assessment and treatment protocols for PwCOPD. They also suggest a role for regulatory and professional bodies in clarifying expectations regarding the training and education required before SLPs deliver EMST in clinical practice. In our sample, most SLPs (71%) reported relying on informal, self-directed learning, while only 29% had completed formal training in EMST. This pattern may reflect limited availability of structured training opportunities, a situation that may have been further influenced by COVID-19-related service disruptions. While informal learning offers flexibility [63], it may not require the demonstration of essential EMST skills, such as manometry techniques and safety considerations. Formal training, by contrast, provides structured content, expert supervision, and standardized competencies, which can enhance confidence [64]. Importantly, reliance on informal training in EMST has potential clinical implications, particularly when working with respiratory-compromised populations such as PwCOPD. Without formal instruction in safety parameters, load titration, and monitoring of respiratory responses, there is a risk of inconsistent or suboptimal implementation. This further highlights the need for accessible, standardized training pathways to ensure safe, confident, and competent EMST delivery to this vulnerable population group.

### 4.3. SLPs Confidence Using EMST with PwCOPD

The predominance of moderate confidence ratings among SLPs when delivering EMST to PwCOPD may reflect several factors. Confidence was not associated with work location, clinical experience, or caseload composition; however, the following points are noteworthy:

1. EMST remains an emerging intervention in routine dysphagia care for PwCOPD rather than an established standard of practice. The emergent rather than established evidence base may contribute to SLP wariness about the use of EMST with PwCOPD, who are an acknowledged vulnerable population group.
2. Reliance on informal training may limit clinicians’ confidence in applying EMST with a medically complex population, which may explain why only 4% of participants in this study reported high confidence levels in the use of EMST with PwCOPD.
3. The analysis of the qualitative data highlighted that some participants (18%) prefer hands-on experience, supervision, and access to specialized resources (e.g., respiratory physiologists and manometry) as key strategies to build confidence. Evidence from other healthcare disciplines shows that structured mentorship enhances knowledge, attitudes, and implementation behaviors, thereby promoting confidence in delivering evidence-based care [65].
4. Education and training, including understanding EMST’s physiological mechanisms, indications, and contraindications, were also highlighted as factors that may enhance clinician confidence when delivering EMST. These findings mirror previous research, which found SLP confidence is shaped not only by knowledge acquisition but also by hands-on experience, supervision, and access to resources [66].

### 4.4. Impact of the COVID-19 Pandemic

Data for this study were collected during the COVID-19 pandemic, a period marked by significant disruption to clinical practice. While 20% of SLPs reported discontinuing EMST for PwCOPD due to concerns regarding aerosol generation, the majority adapted by implementing infection control measures or transitioning to telehealth modalities. This adaptability demonstrates resilience and a commitment to maintaining dysphagia interventions despite heightened risks. This pandemic experience emphasizes the necessity of clear guidelines and safety strategies to support EMST delivery in respiratory-compromised populations during infectious outbreaks. It also highlights the need for improved telehealth readiness to ensure continuity of care when face-to-face therapy is restricted.

The pandemic context yields insights to inform both routine clinical practice and preparedness for future respiratory pandemics. Specifically, standardized clinical guidelines are needed when delivering EMST to PwCOPD, either face-to-face or virtually, including protocols for MEP assessment, monitoring of respiratory responses, and treatment dose adjustments tailored to this population. Strengthening formal training pathways and structured interdisciplinary collaboration are essential to enhance SLPs’ confidence and to ensure consistent implementation of EMST with PwCOPD, particularly by more experienced SLPs. Collaboration with respiratory physiologists and physicians ensures access to objective assessments, facilitates appropriate patient selection, and supports safe integration of EMST into dysphagia management. Embedding EMST within interdisciplinary care, particularly with respiratory physiologists and physicians, would strengthen its implementation and optimize patient safety [58].

### 4.5. Limitations and Future Directions

This study has some limitations. The survey was restricted to SLPs working in dysphagia practice in Ireland, which limits the generalizability of findings to other contexts and geographical regions. The confidence of respondents using EMST with PwCOPD was recorded using self-reported visual analog scales, and perspectives were investigated through a single open-ended question, which may not fully reflect the breadth of clinical experiences. Although this study examined whether SLPs had received formal or informal training prior to delivering EMST in clinical practice, this measure did not allow for an in-depth exploration of the nature or scope of EMST education available to SLPs in Ireland. In addition, the absence of survey questions exploring referral sources of PwCOPD to SLP is a missed opportunity to investigate interdisciplinary aspects of EMST delivery in clinical practice. Despite these limitations, the findings provide valuable insights for researchers interested in exploring SLPs’ use of EMST with PwCOPD, particularly through qualitative approaches that can capture richer detail.

Future research should examine the long-term clinical effectiveness of EMST in PwCOPD, identify factors that facilitate its adoption in routine practice, explore the nature of EMST education and training at undergraduate and postgraduate levels, and refine treatment protocols tailored to this population. The development of telehealth-compatible EMST protocols is also critical to enhance accessibility and ensure continuity of care during healthcare disruptions.

## 5. Conclusions

This study provides the first national overview of how SLPs in Ireland use EMST with PwCOPD. SLPs have high awareness of EMST as a dysphagia intervention for PwCOPD but variable implementation practices. Limited use of objective MEP assessment and reliance on informal training highlight opportunities to strengthen education, standardize protocols for PwCOPD, and enhance interdisciplinary support. Participants report moderate confidence ratings when delivering EMST, shaped by training, experience, and access to specialized respiratory resources. Together, these findings highlight the need for structured training pathways, clearer clinical guidance, and improved resource availability to support safe, consistent, confident, and evidence-based EMST delivery for PwCOPD.

## Figures and Tables

**Table 1:** Work setting of respondents (*n* = 49).

Work Setting	Eligible Sample (%)	No. of Respondents Using EMST (*n*)
Acute Hospital *	65.3%	32
Outpatient/Community Care	18.4%	9
Rehabilitation Services	14.3%	7
Others (Hospice, Nursing Home Care Setting)	2%	1
Total	100%	49

* Most respondents who delivered EMST to people with COPD worked in acute hospital settings.

**Table 2:** Clinical experience of respondents (*n* = 49).

Years of Dysphagia Clinical Experience	Eligible Sample (%)	No. of Respondents Using EMST (*n*)
0–5 years *	26.5%	13
6–10 years *	28.6%	14
11–15 years	18.4%	9
16–20 years	10.2%	5
20 years+	16.3%	8
Total	100%	49

* Over half of the respondents who used EMST with people with COPD had ≤10 years of dysphagia clinical experience.

**Table 3:** Self-reported confidence ratings of respondents when delivering EMST to individuals with COPD and dysphagia.

Confidence Rating (Scores 0–10)	Frequency (*n*)	Percentage (%)
0	1	2.0
1	0	0
2	1	2.0
3	2	4.1
4	3	6.1
5	6	12.2
6 *	11	22.4
7 *	13	26.5
8 *	10	20.4
9	1	2.0
10	1	2.0

* The majority of SLPs reported moderate confidence (scores 6–8), with few reporting high confidence (scores ≥ 9).

**Table 4:** Factors Reported to Improve SLP Confidence in EMST Delivery.

Categories	Respondents (% *n*)	Description of Perceived Enhancers of Confidence	Supporting Quotes from Respondents
Training	57% (*n* = 28)	Structured formal training and supervision by SLPs skilled in EMST: - 1/3 respondents (*n* = 9) suggested practical, face to face delivery. - 4% respondents (*n* = 2) proposed follow up supervision by SLPs (skilled in EMST) to ensure competence.	“My confidence would increase with practical face to face training with guidance on practical use with patients” (Respondent #27). “I would like to work with a Speech and Language Therapist who is very proficient in the use of EMST and manometry for a few days in order to ensure that I am working with it correctly and to see if there are areas I can improve on” (Respondent #47).
Knowledge	10% (*n* =5)	Greater knowledge of the evidence base, physiological impact, indications and contraindications of using EMST with PwCOPD and dysphagia.	“Improved understanding of the physiological impact of EMST and also contraindications” (Respondent #16). “More certainty about if it can be used with patients with poor respiratory baseline” (Respondent #35).
Experience/Exposure	18% (*n* = 9)	Increased frequency and more routine/regular use of EMST with PwCOPD in clinical practice.	“Need to use with more patients to develop competency and confidence” (Respondent #44). “Regular use of EMST. We have a lot of information leaflets in the department, but I think using it regularly and seeing success for patients would help confidence levels” (Respondent #46).
Resource Access	8% (*n* = 4)	Greater access to objective measures of MEP (e.g., manometry) and improved access to respiratory specialists (physicians and respiratory physiologists”.	“I am self-taught and rely heavily on a respiratory physiologist for assessing MEP” (Respondent #11).

## Data Availability

The data that support the findings of this study are openly available in the Zenodo data repository at: https://zenodo.org/records/14229854 (accessed on 3 December 2025), DOI: 10.5281/zenodo.10184770.

## Supplementary Materials

The following supporting information can be downloaded at: https://www.mdpi.com/article/10.3390/jcm15020733/s1, Supplementary S1: Survey Questionnaire Instrument; Supplementary S2: Letter to the Gatekeeper.

## Abbreviations

The following abbreviations are used in this manuscript:

EMST	Expiratory Muscle Strength Training
COPD	Chronic Obstructive Pulmonary Disease
PwCOPD	People Diagnosed with Chronic Obstructive Pulmonary Disease
SLPs	Speech-Language Pathologists
MEP	Maximal Expiratory Pressure
