# Supplementary material for: Expiratory Muscle Strength Training in COPD Dysphagia Management: A Survey of Speech-Language Pathologists
Source: J Clin Med. 2026 Jan 16;15(2):733. doi: 10.3390/jcm15020733 (PMC12841669; doi:10.3390/jcm15020733)
Supplement: Supplementary file 1 [file jcm-15-00733-s001.zip › jcm-4055309-supplementary.pdf]

Supplementary S1: Survey Questionnaire Instrument

"Expiratory Muscle Strength Training in COPD dysphagia management: A survey of Speech-Language Pathologists (SLPs) in the Republic of Ireland"

Part 1: Study Participant Information Leaflet

What is the aim of the research study?

- Explore Speech Language Pathologists (SLPs) awareness, practices and self-reported confidence ratings when using Expiratory Muscle Strength Training (EMST) as a treatment of dysphagia in people with Chronic Obstructive Pulmonary Disease (COPD).
- Investigate if the Covid-19 pandemic has an impact on how SLPs deliver EMST to PwCOPD.

Who can participate?

- Speech Language Pathologists (SLPs) who hold a current registration with CORU (Ireland's multi-profession health & social care regulator).
- SLPs with a professional qualification and clinical experience in assessment and management of dysphagia in adult patients.
- SLPs must be working clinically in the Republic of Ireland.

Who should not participate?

- SLPs who are not currently registered with CORU.
- SLPs who have never worked with adults with dysphagia.
- SLPs who have not worked with adults with dysphagia in the past 3 years.
- SLPs who are working outside the Republic of Ireland.

Do I have to take part?

- No. Survey completion is entirely voluntary.
- You can stop at any stage without giving a reason.
- You will not be contacted following the survey (unless you are the lucky raffle winner- see below).

What happens to my data?

- This survey is fully anonymous.
- Identifying information about you will not be stored and there will be no way for anybody to personally identify you from your responses.
- Anonymised data will be analysed, and the findings will be disseminated via journal publications and/or conferences.

Has this study received ethical approval?

Ethical approval has been received from X.

What is involved?

- Complete an anonymous, online survey which will take approximately 15 minutes to complete.
- You are advised to complete this survey in one sitting.
- <u>Part 2</u> will ask you questions about your demographics and caseload profile.
- <u>Part 3</u> will ask questions about your awareness, practices and confidence using EMST as a dysphagia treatment with people with COPD.

N.B: you may withdraw from the survey at any point. However, following completion of all questions, you will be asked to click on a SUBMIT button. Please note that after clicking SUBMIT, it will not be possible to withdraw the data you have entered in this survey.

How do I provide consent to participate in this study?

If you CONSENT to participate, please click NEXT below.

NEXT

How can I be included in a raffle for a free EMST 150 device?

- At the end of the survey, you will be given the option of entering a raffle. This is entirely optional.
- If you wish to enter, you will be asked to submit a contact email address. This will be used solely for the purposes of contacting you should you be the lucky winner. Contact information will not be assigned to survey data and will be destroyed after the raffle.

Who can I contact if I have queries, complaints or require further information? Primary researcher

PART 2: Demographics and Caseload Profile

Q1. Where do you work?

- Acute hospital o
- Outpatient/ Community based services o
- Rehabilitation services o
- Disability services o
- Other. Please specify: ___________________________ o

Q2. How many years are you working as a Speech & Language Therapist (SLP) in dysphagia?

- 0-5 years o
- 6-10 years o
- 11-15 years o
- 16-20 years o
- 20 years + o

Q3. Approximately, what percentage of your caseload consists of patients with Chronic Obstructive Pulmonary Disease (COPD) and dysphagia?

- 0-25% o
- 26-50% o
- 51-75% o
- 76-100% o

PART 3: Expiratory Muscle Strength Training: Awareness, Confidence and Practices

Q4. Have you heard of Expiratory Muscle Strength Training (EMST) as a dysphagia treatment for people with COPD?

- Yes o
- No o

If you answered No to Q4, then please proceed to Q17.

Q5. Do you use Expiratory Muscle Strength Training to treat dysphagia in people with COPD?

- Yes o
- No o

If you answered No to Q5, then please proceed to Q17.

Q6. Do you have access to Expiratory Muscle Strength Training devices in your work setting?

- Yes o
- No o

Q7. When using Expiratory Muscle Strength Training with people with COPD, how do you assess the participant's maximum expiratory pressure (MEP)? Tick all that apply.

- Using the EMST device (EMST 150 protocol) whereby MEP is defined as the point at which the patient is unable to move air through the device/ open the spring-loaded valve. o
- Hand-held digital manometer e.g., MicroRPN. o
- Respiratory Physiologists assess MEP o

Q8. Do you use any <u>other</u> methods to assess MEP of people with COPD (not outlined in previous question)?

- Yes o
- No o

Q9. If you ticked "Yes" to question 8, please provide details of <u>other</u> methods you use to assess MEP of people with COPD (not mentioned in Q7).

____

____

____

____

Q10. What treatment protocol/regimen do you use when delivering Expiratory Muscle Strength Training to people with COPD? Tick all that apply.

"Rule of Fives" protocol: *Therapy target is set at 75% of the participant's maximal expiratory pressure (MEP). Therapy dosage is 5 sets x 5 reps daily (25 reps daily) x 5 days per week x 5 weeks.* o

Modified EMST protocol based on the client's presentation o

Q11. Please provide any further details of the treatment protocol you use when delivering EMST to people with COPD.

____

____

Q12. Has Covid-19 impacted on your use of EMST as part of dysphagia therapy with individuals with COPD? Please tick all statements that apply.

- I avoid using EMST because it is an aerosol generating procedure o
- I deliver EMST face to face but follow infection control guidelines for aerosol generating procedures o
- I deliver EMST using teletherapy o
- I deliver EMST using a combination of face to face and teletherapy o

Q13. What training did you undertake before using Expiratory Muscle Strength Training in clinical practice?

- Self-taught o
- Trained by another member of the SLP Team o
- Trained by another discipline e.g., physiotherapist or doctor o
- Formal online training o
- Formal face to face workshop training o
- Other o

Q14. If you ticked "Other "for question 13, please provide details of other training (not mentioned in Q13) undertaken before using Expiratory Muscle Strength Training (EMST)?

Q15. How confident are you to conduct Expiratory Muscle Strength Training (EMST) as a dysphagia treatment approach to people with COPD?

Very Confident -- Not confident

Q16. How do you think your confidence could be improved when delivering Expiratory Muscle Strength Training (EMST) to PwCOPD?

Q17. "I am interested in increasing my knowledge in the area of Expiratory Muscle Strength Training as a dysphagia treatment approach"?

- Yes o
- No o

CONSENT

By clicking the **SUBMIT** button below, you indicate that you:

- consent to the collection of the information you have provided; **and**
- consent to the use of this anonymized data for analysis **and** for dissemination of the findings in journal publications and/or conference presentations.

Please note that after clicking SUBMIT, it will not be possible to withdraw the data you have entered in this survey.

SUBMIT

Supplementary S2: Letter to the Gatekeeper:

"Expiratory Muscle Strength Training in COPD dysphagia management: A survey of SLPs in the Republic of Ireland."

Dear Colleague,

My name is X. I am a Senior Speech and Language Therapist and researcher at the University of Galway.

I am seeking participants for this research study "Expiratory Muscle Strength Training in COPD dysphagia management: A survey of SLPs in the Republic of Ireland ".

Purpose of the research study:

What is the aim of the PhD research?

- Explore Speech and Language Therapists (SLPs) awareness, practices and self-reported confidence ratings when using Expiratory Muscle Strength Training (EMST) as a treatment of dysphagia in people with Chronic Obstructive Pulmonary Disease (COPD).
- Investigate if the Covid-19 pandemic has an impact on how SLPs deliver EMST to PwCOPD.

Who can participate?

- Speech Language Pathologists (SLPs) who hold current registration with CORU (Ireland's multi-profession health & social care regulator).
- SLPs with a professional qualification and clinical experience in assessment and management of dysphagia in adult patients.
- SLPs must be working clinically in the Republic of Ireland.

What is involved?

- Participants will be asked to complete an anonymous, online survey which will take approximately 15 minutes to complete.
- All participants who complete this survey have the option of entering a raffle for a free EMST150 device. If they wish to be included, they are asked to include a contact number or email address. This contact information will be used only for the purposes of contacting the lucky winner and will be destroyed after the raffle.
- If you wish to participate in this survey, please click on the link below: https://forms.office.com/Pages/ResponsePage.aspx?id=hrHjE0bEq0qcbZq5u3aBbPN-3Nd6DzBIrK9xjz0-TTRURU1ENDJaVkYzQ1VKN1ZXUkRMU09LVEU3Ti4u
- **NB: Please submit only ONE response to this survey to prevent duplicate entries.**

Contact Details: If you have any queries, complaints or require any further information regarding this survey, please contact: Primary Investigator @email address.
